# Grid Search and Genetic Algorithm Optimization of Neural Networks for Automotive Radar Object Classification †

**DOI:** 10.3390/s25196017

**Published:** 2025-09-30

**Authors:** Atila Gabriel Ham, Corina Nafornita, Vladimir Cristian Vesa, George Copacean, Voislava Denisa Davidovici, Ioan Nafornita

**Affiliations:** 1 Communications Department, Politehnica University of Timisoara, 300223 Timisoara, Romania; voislava.davidovici@student.upt.ro (V.D.D.); ioan.nafornita@upt.ro (I.N.); 2 Hella Forvia Romania, 300011 Timisoara, Romania; vladimircristian.vesa@forvia.com (V.C.V.); george.copacean@forvia.com (G.C.)

**Keywords:** autonomous driving, automotive, radar, object classification, neural network, grid search, genetic algorithm

## Abstract

This paper proposes and evaluates two neural network-based approaches for object classification in automotive radar systems, comparing the performance impact of grid search and genetic algorithm (GA) hyperparameter optimization strategies. The task involves classifying cars, pedestrians, and cyclists using radar-derived features. The grid search–optimized model employs a compact architecture with two hidden layers and 10 neurons per layer, leveraging kinematic correlations and motion descriptors to achieve mean accuracies of 90.06% (validation) and 90.00% (test). In contrast, the GA-optimized model adopts a deeper architecture with nine hidden layers and 30 neurons per layer, integrating an expanded feature set that includes object dimensions, signal-to-noise ratio (SNR), radar cross-section (RCS), and Kalman filter–based motion descriptors, resulting in substantially higher performance at approximately 97.40% mean accuracy on both validation and test datasets. Principal Component Analysis (PCA) and SHapley Additive exPlanations (SHAP) highlight the enhanced discriminative power of the new set of features, while parallelized GA execution enables efficient exploration of a broader hyperparameter space. Although currently optimized for urban traffic scenarios, the proposed approach can be extended to highway and extra-urban environments through targeted dataset expansion and developing additional features that are less sensitive to object kinematics, thereby improving robustness across diverse motion patterns and operational contexts.

## 1. Introduction

Autonomous driving demands that vehicles equipped with radar sensors and other sensing modalities not only perceive their surroundings, offering precise detection [1], but also interpret and understand the environment in a meaningful way. A critical component of this understanding is the classification of surrounding moving objects, which plays a key role in enabling safe and effective navigation in complex traffic scenarios. Several studies have addressed this problem, particularly within the context of radar-based object recognition. Traditional approaches to target recognition using radar data typically involve the extraction of hand-crafted features followed by classification through machine learning techniques such as Support Vector Machines (SVM) [2,3,4]. However, these approaches often lacked robustness in dynamic scenarios. More recently, methods based on neural networks and deep learning have gained attention due to their ability to learn complex patterns directly from data. In ref. [5], the authors introduced a deep learning-based classifier that processes individual radar reflections, each characterized by attributes such as range, radial velocity, radar cross section, and azimuth angle. Another novel approach is presented in ref. [6], where the authors apply spiking neural networks (SNNs) for the classification of radar-detected objects in automotive contexts. Furthermore, ref. [7] proposes a methodology based on Long Short-Term Memory (LSTM) layers. This method involves transforming raw data into a unified coordinate system, performing grouping and labeling, and subsequently applying the classification model. An additional challenge in radar-based object classification is the imbalance of classes, where certain object types (e.g., pedestrians or cyclists) are underrepresented compared to others (e.g., cars). To mitigate this issue, oversampling techniques such as the Synthetic Minority Over-sampling Technique (SMOTE) [8] have been widely used in machine learning to generate synthetic samples for minority classes, thereby improving classifier performance in imbalanced datasets. Using this technique [8], in ref. [9] we proposed the use of neural networks to classify targets using attributes about the objects such as their trajectory, acceleration, velocity, standard deviation of velocity and acceleration, object length and width and other parameters related to object’s movement. Selection of hyperparameters is made by using grid search.

Building on this foundation, this paper proposes two neural network-based methods for classifying moving objects detected by automotive radar systems in urban environments. We focus on classification of three main object categories: pedestrians, cyclists, and cars. While previous work such as Ulrich et al. [5] relies on features extracted from a fixed number of individual radar reflections, our method aggregates reflections to compute statistical descriptors, such as means and variances, offering a more stable representation of object-level characteristics. Hyperparameter optimization is done using GridSearch and the genetic algorithm, respectively. The following are the paper’s main contributions:

- Creation and labeling of an expanded real-world radar dataset for three object classes: car, pedestrian, and bicycle, in urban scenarios;
- Selection of appropriate features based on ranking and Pearson correlation analysis, respectively;
- Proposal, implementation and validation of a neural network classifier with grid search-based hyperparameter tuning, extending the method in [9];
- Proposal, implementation and validation of a new classifier using genetic algorithms for hyperparameter optimization.

Implementation details of the proposed models, as well as the evaluation procedure, are discussed in subsequent sections. The final part of the paper presents experimental results demonstrating the efficiency of the proposed classification methods, followed by conclusions and future work.

## 2. Materials and Methods

In this study, we employed the same neural network architecture and feature set as presented in [9]; however, our approach now uses a substantially larger dataset, which enables a more comprehensive evaluation of model performance. Additionally, we introduced new metrics to measure the performance of the neural network. This enhancement allows for a more robust and nuanced analysis compared to the original framework. The proposed approach utilizes features derived from tracked objects, specifically width, length, speed, acceleration, and the standard deviation of both speed and acceleration, radar cross section (RCS) and signal-to-noise ratio (SNR). All features, with the exception of the standard deviation of the object’s position along the X and Y axes, are expressed in terms of ground-relative velocity and not relative velocity. Feature selection is critical to ensure effective discrimination among object classes; in this context, velocity is considered a particularly informative feature. Radar reflections associated with each object are aggregated, and features are computed as statistical descriptors (e.g., mean SNR, variance in speed) over all available reflections. This contrasts with approaches that use a fixed number of raw reflections per object [5], which may introduce variability due to noise or partial detection. 

To optimize the performance of the neural network classifier, we introduced a new hyperparameter search strategy, which is the genetic algorithm.

### 2.1. Grid Search Hyperparameter Optimization Technique

Grid search is a systematic and exhaustive hyperparameter optimization technique commonly employed in machine learning to enhance model performance. It operates by defining a discrete set of values for each hyperparameter and evaluating the model performance for every possible combination within this predefined grid. Each combination is assessed using a performance metric, typically through cross-validation, to ensure the robustness and generalizability of the results. This method is particularly useful when the hyperparameter space is relatively small and the computational resources are sufficient to support the exhaustive search. Although simple and effective, grid search is computationally expensive compared to other methods [10], especially as the number of hyperparameters and their respective candidate values increase. This makes it less suitable for high-dimensional or large-scale problems. Nevertheless, grid search remains a foundational technique in hyperparameter tuning due to its deterministic nature and its ability to guarantee the identification of the optimal configuration within the specified grid. It has also been used in automotive radar classification, see for example [9].

### 2.2. Genetic Algorithm Hyperparameter Optimization Technique

We also propose the use of genetic algorithms for the hyperparameter search, as an alternative method. Genetic algorithms (GAs) have been proposed as “computer programs that evolve in ways that resemble natural selection” [11]. Populations of potential solutions evolve through selection, crossover, and mutation to optimize complex problems. These algorithms mimic biological evolution by iteratively refining candidate solutions based on their fitness, making them particularly effective for optimization tasks in engineering, artificial intelligence, and machine learning [12]. In neural networks, GAs can be used to optimize the neural network architecture (e.g., the optimal number of layers, neurons per layer, and connectivity patterns), tune hyperparameters (e.g., to adjust learning rates, activation functions, and other training parameters) or select features (identify the most relevant input features for a given application).

GAs have also been used in automotive, for optimization, machine learning, sensor fusion, testing and validation [13,14,15,16,17,18,19]. In ref. [14], the DeepHybrid Model integrates both radar spectra and reflection attributes as inputs to a deep neural network, for the purpose of object classification. The authors apply an automatic search of the neural network architecture (NAS) to identify a resource-efficient network while maintaining high performance. They use a combination of the non-dominant sorting genetic algorithm II and the regularized evolution algorithm. The study [16] explores the use of GAs to optimize both the region of interest from radar signals and the classifier’s architecture, increasing detection precision and hardware efficiency.

### 2.3. Radar Signal Processing Pipeline and Workflow of the Methods

**Figure 1:**
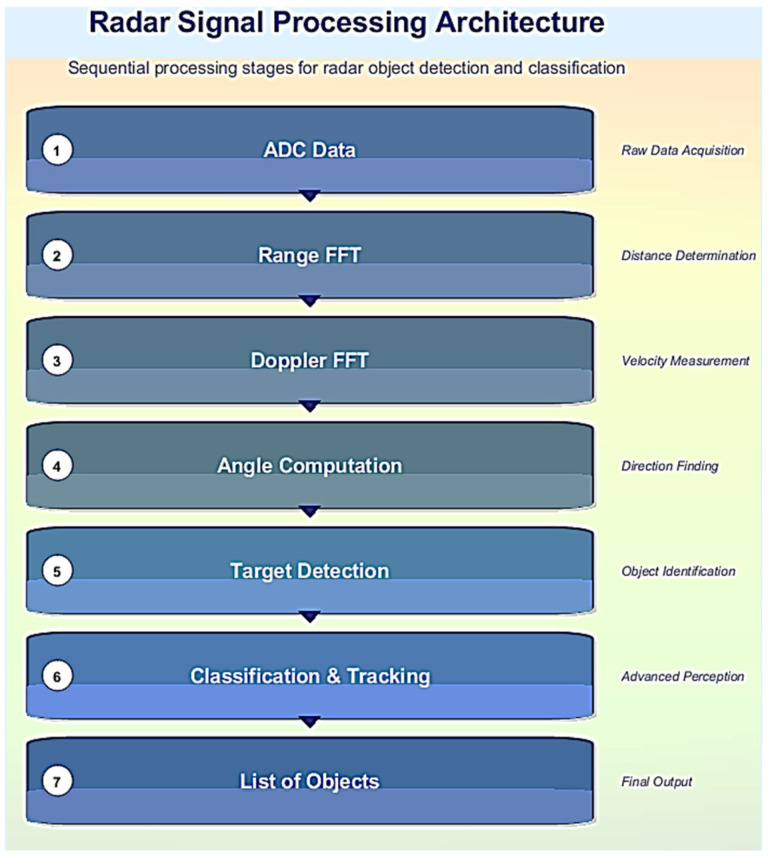
Radar signal processing architecture.

The conventional automotive radar signal processing architecture is shown in Figure 1. It consists of the following stages:

- Signal Reception and Digitization: Reflected radar signals are received by the antenna and converted into digital form, resulting in a 3D raw data cube (samples × chirps × antennas).
- Range FFT: A Fast Fourier Transform (FFT) is applied along the range dimension to extract distance information.
- Doppler FFT: A second FFT is applied along the Doppler dimension to estimate object velocity based on frequency shifts.
- Angle Calculation: Techniques such as beamforming or MUSIC are used to determine azimuth and elevation angles from phase differences across antennas.
- Target Detection: Constant False Alarm Rate (CFAR) algorithms [20] are applied to identify potential targets while maintaining low false alarm rates.
- Classification and Tracking: Detected objects are classified using machine learning models and tracked using algorithms such as Kalman filters [21] or particle filters [22]. Data particle-based methods have also been applied in radar signal recognition [23], which can complement traditional tracking techniques.
- Object List Generation: The final output includes object positions (x, y, z), velocities, sizes, and other relevant attributes for downstream tasks such as collision avoidance and adaptive cruise control.

The Classification & Tracking stage involves classifying detected targets based on their characteristics, such as size or speed, using machine learning algorithms or rule-based methods. Tracking algorithms predict future positions by considering past measurements, ensuring continuous monitoring even if temporary occlusions occur. This stage is crucial for maintaining a consistent track of objects over time, which is important for applications like autonomous driving where real-time decision-making is required.

The algorithms proposed are in the Classification & Tracking stage of the radar signal processing architecture. In the radar signal processing architecture (Figure 1), the features employed for the proposed object classification methods originate from two principal stages of the signal processing pipeline. The first stage involves the extraction of features from raw targets, which are the immediate outputs of the radar signal processing block. These raw targets represent individual radar reflections and are not yet associated with specific objects. Features derived at this level are typically low-level descriptors that capture the physical and signal-related properties of the radar returns. Examples include the range variance of raw targets, mean signal-to-noise ratio (SNR) of raw targets, speed variance of raw targets, Y position variance of raw targets, and the variance in SNR. Additionally, spatial descriptors such as the length and width of the radar target cloud hypothesis, as well as the filtered radar cross section (RCS), are also computed at this stage.

The second stage pertains to the tracking component, where raw targets are aggregated and processed to form coherent object-level representations. This process typically involves the application of filtering techniques, such as Kalman filters, to estimate the kinematic and geometric properties of moving objects over time. Features derived from this stage are of higher-level and provide temporally stable information that reflects the dynamics and structure of the tracked objects. These include the overground acceleration hypothesis, Kalman filter-based variances in heading, X-axis and Y-axis velocities, and yaw rate. Furthermore, features such as the overground speed hypothesis, object length hypothesis, and object area hypothesis are also computed based on the tracking output. These features are particularly valuable in urban driving scenarios, where accurate modeling of object behavior is essential for reliable classification.

**Figure 2:**
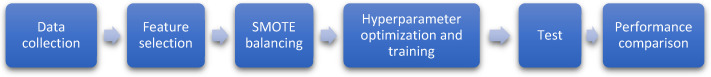
Workflow diagram.

The working flow diagram of the proposed methods is shown in Figure 2.

The first step is to collect data about the object interest class: cars, pedestrians and bicycles. This will be accomplished by re-simulating real urban traffic scenarios using Software-in-the-Loop (SIL) methodology.

The second step involves extracting the most relevant features from the object. For the first method, this is done using ranking, while for the second method, this was achieved by analyzing the correlation matrix using Pearson’s correlation coefficient, which measures a linear correlation between two vectors:

(1) $$r_{xy}=\frac{\sum_{i=1}^{n}\left(x_{i}-\bar{x}\right)\left(y_{i}-\bar{y}\right)}{\sqrt{\sum_{i=1}^{n}{\left(x_{i}-\bar{x}\right)}^{2}}\sqrt{\sum_{i=1}^{n}{\left(y_{i}-\bar{y}\right)}^{2}}},$$

where *n* is sample size; *x_i_* and *y_i_* are the individual sample points indexed with i , $\bar{x}$ and $\bar{y}$ are the sample means of the vectors.

The third step consists of synthetic data generation to address class imbalance, using SMOTE, as described in the next section. The fourth step involves identifying the optimal combination of hyperparameters and training the neural networks. For the first method, hyperparameter optimization is achieved using GridSearch, similar to the work in [9], and for the second method proposed, it is achieved using a genetic algorithm. Our implementation of the genetic algorithm explored various configurations of batch size, epochs, optimizer, learning rate, hidden units, and the number of layers. To enhance efficiency and reduce time consumption, the genetic algorithm was executed in parallel. This approach allowed for a comprehensive search of the hyperparameter space, ensuring the selection of the most effective parameters for the model.

The fifth part involved testing the neural network in terms of the following parameters: accuracy, precision, recall, F1 score for each class, as well as micro-average parameters. Precision is the fraction of detections reported by the model that were correct:

(2) $$\mathrm{Precision}=\frac{tp}{tp+fp},$$

Precision for a given class is defined similarly, as the ratio of instances correctly classified in that class and all instances the model predicted to belong to that class:

(3) $${\mathrm{Precision}}_{\mathrm{class}}=\frac{\mathrm{tp\_class}}{\mathrm{tp\_class}+\mathrm{fp\_class}},$$

The recall, also known as the true positive rate TPR or sensitivity, is the fraction of true events that were detected:

(4) $$\mathrm{Recall}=\mathrm{Sensitivity}=\frac{tp}{tp+fn},$$

We also use recall for each class, which is the ratio of the sum of true positives for a certain class, divided by the sum of all True Positives and False Negatives in the data:

(5) $${\mathrm{Recall}}_{\mathrm{class}}=\frac{\mathrm{tp\_class}}{\mathrm{tp\_class}+\mathrm{fn\_class}},$$

The F1 score is the harmonic mean of precision *p* and recall *r*:

(6) $$F1\;score=\frac{2\cdot \mathrm{Precision}\cdot \mathrm{Recall}}{\mathrm{Precision}+\mathrm{Recall}}=\frac{2\cdot tp}{2tp+fp+fn},$$

Accuracy represents the ratio of correctly classified instances, both positive and negative, to the total number of evaluated instances:

(7) $$\mathrm{Accuracy}=\frac{tp+tn}{tp+ep+tn+fn}$$

Other metrics are macro-average and micro-average precision and recall, respectively. To obtain the macro-average precision and recall, the precision and recall across all classes are averaged. These metrics do not account for class imbalance.

(8) $$\mathrm{Macro}-\mathrm{average}\;\mathrm{Precision}=\frac{\mathrm{Precision\_class1}+\cdots +\mathrm{Precision\_class}N}{N},$$

(9) $$\mathrm{Macro}-\mathrm{average}\;\mathrm{Recall}=\frac{\mathrm{Recall\_class1}+\cdots +\mathrm{Recall\_class}N}{N},$$

The micro-average precision and recall take into account contributions from all classes. For example, the micro-average precision is the total number of true positives across all classes divided by the total number of predicted positives across all classes, while micro-average recall is the total number of true positives across all classes divided by the total number of actual positives across all classes. Since these metrics treat each prediction individually, regardless of class, they are suitable for imbalanced classes. They are equivalent to the macro-average metrics due to the application of the SMOTE, which results in a balanced dataset.

(10) $$\mathrm{Micro}-\mathrm{average}\;\mathrm{Precision}=\frac{\mathrm{tp\_class1}+\cdots +\mathrm{tp\_classN}}{\mathrm{tp\_class1}+\mathrm{fp\_class1}+\cdots +\mathrm{tp\_classN}+\mathrm{fp\_classN}},$$

(11) $$\mathrm{Micro}-\mathrm{average}\;\mathrm{Recall}=\frac{\mathrm{tp\_class1}+\cdots +\mathrm{tp\_classN}}{\mathrm{tp\_class1}+\mathrm{fn\_class1}+\cdots +\mathrm{tp\_classN}+\mathrm{fn\_classN}}$$

The last two steps are model evaluation on the testing dataset using the aforementioned performance metrics, as well as a performance comparison to analyze the trade-offs between model complexity, accuracy, and deployment feasibility.

## 3. Implementation

In this section, we will present the implementation of the two methods proposed.

### 3.1. Data Collection (Dataset)

The dataset is integral to the training process of the neural network and has a significant impact on the final decision-making. Our dataset comprises measurements from real objects, collected during instances when the host vehicle was navigating freely within an urban environment. In contrast to the methodology presented in our previous work [9], “Radar Object Classification Using Neural Networks in an Urban Automotive Scenario,” the current paper employs a single, consolidated dataset sourced from urban driving environments. This dataset has been substantially expanded and is now partitioned into 60% for training, 20% for validation, and 20% for testing. Relative to the earlier implementation, the training subset has increased by a factor of 5694.65, while the validation and testing subsets have each grown by a factor of 5695.708. This significant augmentation in dataset size is anticipated to enhance the generalization capability of the neural network model. In the first method, a total of 70 features were extracted based on their potential to broadly characterize the properties of detected objects. These features were selected to capture various aspects of object geometry, motion, and radar signal response. In contrast, the second method involved the design of a distinct set of 32 features aimed at enhancing the representation of object characteristics. These features were specifically tailored to capture the most relevant descriptors for object classification, with a focus on improving both model performance and computational efficiency.

**Figure 3:**
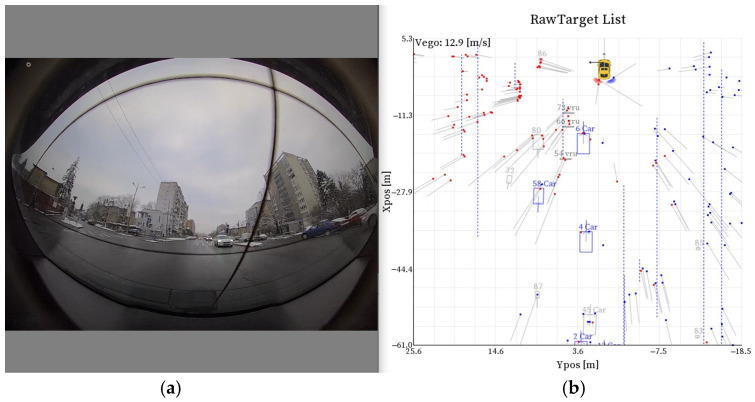
Urban street scene captured with a fisheye camera (**a**) and corresponding object tracking and classification results (**b**), showing detected targets plotted in the X-Y position space.

In the first case, we used ranking to select the most important features, as shown in Section 3.2. In the second case, we selected those that pertain to the object’s movement, size, SNR, and RCS, as shown in Section 3.3. These selected features are crucial for accurately modeling and predicting the object’s behavior and characteristics. Figure 3 illustrates one frame from our recordings, providing a view of the urban environment and the corresponding radar data.

Figure 3a shows a frame from our recordings, with Figure 3b showing various objects with labels attached to them. Additionally, reflections from the surrounding environment are visible. This image provides a detailed representation of the urban scenario, combining visual data with analytical annotations to enhance the understanding of object detection and classification in complex environments. The labeled objects and environmental reflections contribute to a comprehensive analysis, facilitating the evaluation of the radar system’s performance in real-world scenarios. Our dataset is stored in a comma-separated values (CSV) format.

### 3.2. Feature Selection for the Grid Search Method

To facilitate the selection of relevant features from a dataset, a Python 3.9.13-based analytical script was developed in ref. [9]. The primary objective of this script is to identify and retain only those features that contribute meaningfully to the classification task, while discarding those that are predefined or irrelevant, such as object age or the number of radar cycles. The script begins by reading the dataset and applying a normalization technique using the MinMaxScaler. This transformation scales all feature values to a range between 0 and 1, thereby ensuring that the neural network does not disproportionately favor features with inherently larger numerical values.

Subsequently, the script evaluates feature importance by analyzing the statistical distribution of each feature across different object classes. For each class, the mean value of every feature is computed. The absolute differences between the mean of a given class and the means of the other classes are then calculated. Given the presence of three classes: pedestrian (class 1), cyclist (class 3), and car (class 5), three pairwise comparisons are performed: 1–3, 1–5, and 3–5. The mean of these three absolute differences is then computed for each feature, providing a scalar measure of its discriminative power across all classes.

For interpretability, the resulting mean values are scaled to a range of 0 to 100, yielding a normalized feature importance score. Additionally, a ranking mechanism is applied based on these scores to further elucidate the relative contribution of each feature to the classification task. 

For instance, the feature “width” has a low absolute mean difference of 5.18 between classes 1 and 3, indicating limited utility in distinguishing pedestrians from cyclists. However, the same feature shows significantly higher differences in the 1–5 (142.05) and 3–5 (136.87) comparisons, suggesting its effectiveness in differentiating cars from the other two classes. Despite this, the overall rank of “width” is relatively low (rank 44), implying that other features, such as “length” (rank 8), offer more consistent discriminative power across all class comparisons. An example can be observed in Table 1, which is a fragment of the original table, an excerpt from the resulting table used for illustrative purposes.

After applying the above method, the following 29 features were found:

○ corrXAccelerationAbsYAccelerationAbs, the correlation between the absolute accelerations along the X and Y axes. It provides information about coordinated movement patterns of the object in the horizontal plane.
○ corrXPosXAccelerationAbs, the correlation between the X-position and the absolute X-acceleration of the object. It is useful for understanding how positional changes relate to acceleration behavior along the longitudinal axis.
○ corrXPosXVelocityAbs, the correlation between the X-position and the absolute X-velocity. It helps assess the consistency of motion along the X-axis.
○ corrXPosYPos, the correlation between the X and Y positions of the object, offering insights into the trajectory and directional movement.
○ corrXVelocityAbsXAccelerationAbs, the correlation between absolute X-velocity and absolute X-acceleration, which is important for analyzing acceleration trends relative to speed.
○ corrXVelocityAbsYVelocityAbs, the correlation between absolute velocities along the X and Y axes, indicating the movement in the longitudinal and lateral space for the object.
○ corrYPosYAccelerationAbs, the correlation between Y-position and absolute Y-acceleration, providing information on lateral motion dynamics.
○ corrYPosYVelocityAbs, the correlation between Y-position and absolute Y-velocity, which is useful for evaluating lateral movement consistency.
○ corrYVelocityAbsYAccelerationAbs, the correlation between absolute Y-velocity and absolute Y-acceleration, offering information about lateral acceleration behavior.
○ height: This feature denotes the estimated height of the object, contributing to its geometric characterization.
○ stdDevHeight, the standard deviation of the object’s height, indicates variability in vertical dimensions and potential object instability.
○ stdDevLength, the standard deviation of the object’s length, which reflects changes in perceived object size due to motion.
○ stdDevWidth, the standard deviation of the object’s width, provides information about the changes in object’s geometric configuration.
○ stdDevXAccelerationAbs, the variability in absolute X-acceleration, which is important for assessing dynamic behavior along the longitudinal axis.
○ stdDevXPos, the standard deviation of the X-position, indicating the spread or uncertainty in longitudinal positioning.
○ stdDevXVelocityAbs, the standard deviation of absolute X-velocity, offering information about speed consistency along the X-axis.
○ stdDevYAccelerationAbs: This feature measures the variability in absolute Y-acceleration, which is relevant for analyzing lateral dynamic behavior.
○ stdDevYPos: This feature captures the standard deviation of the Y-position, indicating lateral positional dispersion.
○ stdDevYVelocityAbs: This feature represents the standard deviation of absolute Y-velocity, providing information on lateral speed variability.
○ xAccelerationAbs: This feature denotes the absolute acceleration along the X-axis, which is useful for understanding way of motion.
○ xVelocityAbs: This feature captures the absolute velocity along the X-axis, representing the object’s longitudinal speed.
○ yAccelerationAbs: This feature denotes the absolute acceleration along the Y-axis, which is important for lateral movement analysis.
○ yVelocityAbs: This feature represents the absolute velocity along the Y-axis, indicating the object’s lateral speed.
○ length: This feature estimates the length of the object, contributing to its geometrical characterization.
○ width: This feature denotes the width of the object, providing information about the object’s width.
○ xyAccAbs: This feature represents the combined absolute acceleration in the X and Y directions, providing a view of the object’s dynamic behavior.
○ xyVelAbs: This feature captures the combined absolute velocity in the X and Y directions, offering a measure of object speed.
○ stdDevxyAccAbs: This feature measures the standard deviation of the combined absolute acceleration, indicating variability in overall dynamic behavior.
○ stdDevxyVelAbs: This feature represents the standard deviation of the combined absolute velocity, providing insights into the consistency of the object’s overall motion.

Features such as the “Object Classification Hypothesis” and “Object ID Hypothesis” were excluded because they do not provide informative value for the analysis but are instead used as identifiers for the object ID and the object class.

### 3.3. Feature Selection for the Genetic Algorithm Method

Reducing the number of input features is an important consideration in the design of neural network models, as it enables the use of deeper and more complex architectures while maintaining computational efficiency. A more compact feature set decreases the dimensionality of the input space, thereby reducing the number of operations required per inference and minimizing memory consumption. For the Genetic algorithm-based method, we focus on utilizing a reduced set of 16 features, selected based on their ability to effectively characterize and discriminate between object classes. This approach enhances the computational performance of the model and also contributes to improved classification accuracy by eliminating redundant or non-informative inputs. Consequently, the network can allocate more representational capacity to learning salient patterns relevant to the classification task.

For the feature selection process, we analyzed the correlation matrix using Pearson’s correlation coefficient. Pearson’s technique measures the linear relationship between two variables, providing a value between −1 and 1. A value of 1 indicates a perfect positive linear relationship, −1 indicates a perfect negative linear relationship, and 0 indicates no linear relationship. This method is instrumental in identifying and selecting features that have significant correlations, thereby enhancing the predictive power of the model.

**Figure 4:**
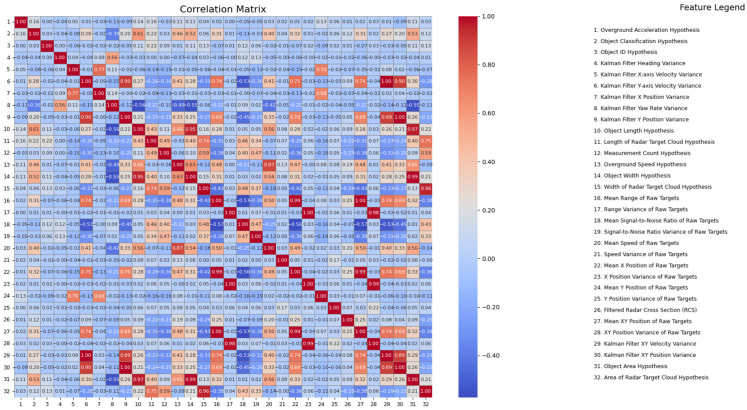
Correlation matrix.

Figure 4 represents the correlation matrix for all features in our dataset. Just like in method 1, features such as the “Object Classification Hypothesis” and “Object ID Hypothesis” were excluded. It is evident that some features exhibit strong correlations, indicating redundancy. Redundant features do not contribute new information to the model, as they are highly correlated with other features. In this phase of the analysis, feature selection was conducted by identifying and retaining those that have minimal redundancy with other features. This approach allows for a more nuanced selection process, emphasizing interpretability and predictive value. As a result, the following 16 features were selected, among which 3 of them are common to the previous method (xyAccAbs with Overground Acceleration Hypothesis, xyVelAbs with Overground Speed Hypothesis, length with Object Length Hypothesis):

- Overground Acceleration Hypothesis: This feature represents the acceleration of the object over the ground. It is crucial for understanding the dynamics and movement patterns of the object.
- Kalman Filter Heading Variance: This feature measures the variance in the object’s heading as estimated by the Kalman filter. It is important for assessing the stability and accuracy of the object’s directional movement.
- Kalman Filter X-axis Velocity Variance: This feature captures the variance in the object’s velocity along the X-axis, providing insights into the object’s speed and movement consistency.
- Kalman Filter Y-axis Velocity Variance: Similarly to the X-axis velocity variance, this feature measures the variance in the object’s velocity along the Y-axis.
- Kalman Filter Yaw Rate Variance: This feature represents the variance in the object’s yaw rate, which is essential for understanding rotational movements and changes in direction.
- Object Length Hypothesis: This feature estimates the length of the object, which is important for size characterization and spatial analysis.
- Length of Radar Target Cloud Hypothesis: This feature measures the length of the radar target cloud, providing information on the spatial extent of the detected object.
- Overground Speed Hypothesis: This feature represents the object’s speed over the ground, which is crucial for movement analysis and prediction.
- Width of Radar Target Cloud Hypothesis: This feature measures the width of the radar target cloud, contributing to the spatial characterization of the object.
- Range Variance of Raw Targets: This feature captures the variance in the range measurements of raw targets, providing insights into the object’s distance and positional accuracy.
- Mean Signal-to-Noise Ratio of Raw Targets: This feature represents the average signal-to-noise ratio of raw targets, which is important for assessing the quality and reliability of the radar data.
- Speed Variance of Raw Targets: This feature measures the variance in the speed of raw targets, contributing to the analysis of movement consistency.
- Y Position Variance of Raw Targets: This feature captures the variance in the Y position of raw targets, providing spatial accuracy information.
- Filtered Radar Cross Section (RCS): This feature represents the filtered radar cross section of the object, which is crucial for understanding the object’s reflectivity and radar signature.
- Object Area Hypothesis: This feature estimates the area of the object, contributing to size characterization and spatial analysis.
- Signal-to-Noise Ratio Variance of Raw Targets: This feature measures the variance in the signal-to-noise ratio (SNR) of raw targets. SNR is a critical metric in radar systems, as it quantifies the ratio of the signal power to the noise power. A higher SNR indicates a clearer and more distinguishable signal from the background noise. The variance in SNR provides insights into the consistency and reliability of the radar measurements, which is essential for accurate object detection and tracking.

**Figure 5:**
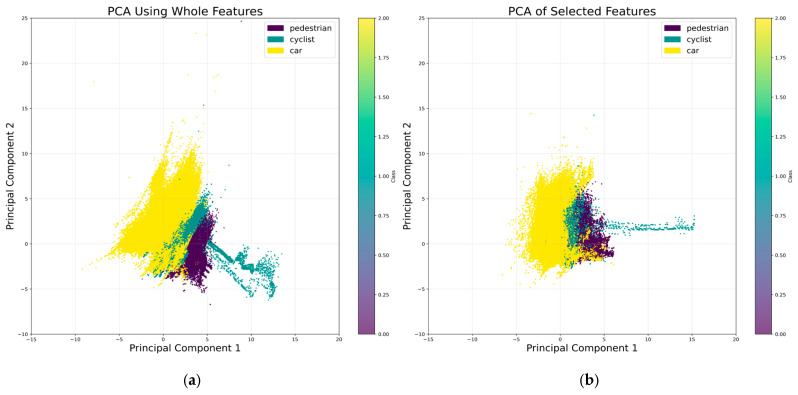
PCA plot: (**a**) for the whole dataset, (**b**) for the selected features used in NN.

Figure 5 shows the distribution of classes within the whole dataset across all the features, utilizing Principal Component Analysis (PCA). PCA is a statistical technique that transforms the original features into a set of linearly uncorrelated components, ordered by the amount of variance they capture. This method is particularly advantageous for data visualization as it reduces dimensionality while preserving the most significant variance in the data. By visualizing the data in this reduced space, we can better understand the separation of classes, which is crucial for evaluating the performance of future neural network models. Effective class separation in the PCA-transformed space often correlates with improved model performance, as it indicates that the features are informative and discriminative.

Figure 5 presents the PCA plot for the features utilized in the neural network. The analysis of the two PCA plots in Figure 5 reveals distinct patterns in the distribution of classes, which are expected to influence the performance of the neural network on the test dataset:

- Car Class: The car class exhibits a well-defined distribution, as evidenced by the clear separation in the PCA plots. This distinct clustering suggests that the features associated with cars are highly informative and discriminative. Consequently, the neural network is likely to achieve high performance in classifying cars within the test dataset.
- Cyclist Class: The cyclist class demonstrates a poorly defined distribution, with significant overlap and intercalation with the pedestrian and car classes. This lack of clear separation indicates that the features for cyclists are less distinctive, leading to potential misclassification. As a result, the neural network may struggle to accurately distinguish cyclists from other classes, negatively impacting its performance on the test dataset. However, the distribution is improved after reducing the number of features—see Figure 5b.
- Pedestrian Class: The pedestrian class shows a better-defined distribution compared to the cyclist class, with slightly improved separation from the other classes. This suggests that the features for pedestrians are more consistent and discriminative, albeit not as distinct as those for cars. Therefore, the NN is expected to perform moderately well in classifying pedestrians, with better results than for cyclists but not as high as for cars.

**Figure 6:**
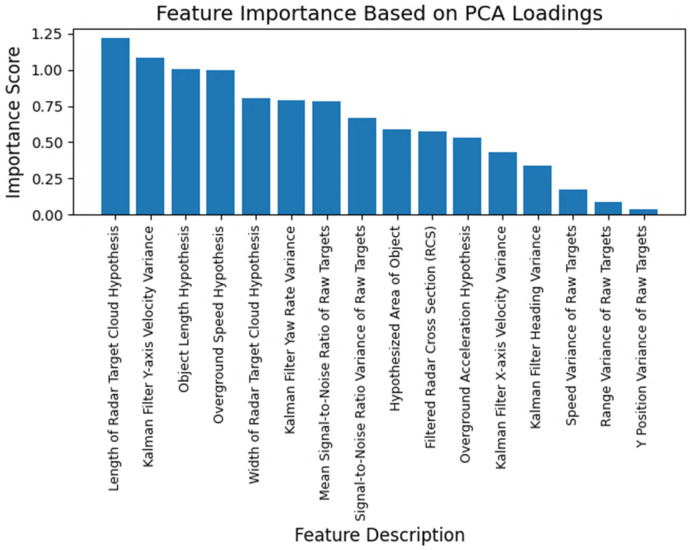
PCA Feature Importance.

Figure 6 illustrates the importance of features utilized in the neural network, as determined by Principal Component Analysis (PCA) loadings. PCA loadings represent the coefficients of the original features in the principal components, indicating how much each feature contributes to the variance captured by the components. Features with larger absolute values of loadings are considered more important because they contribute more significantly to the principal components. For example, the length of the radar target cloud (RTC) is a highly significant feature in the context of PCA due to its direct influence on the variance structure of radar-derived datasets. The RTC length serves as a key descriptor because it captures the spatial extent of the radar returns corresponding to a target object. One of the primary reasons for RTC length’s importance lies in its inherent variability across different objects. Since objects vary considerably in their physical dimensions and geometric shapes, the length of their corresponding radar target clouds also varies significantly. This variation effectively reflects intrinsic differences among objects, making RTC length a powerful feature for distinguishing between target classes. From a practical standpoint, the ability to measure the length of the radar target cloud provides an intuitive and straightforward approach for categorizing objects. Length is a fundamental geometric attribute that often correlates with the physical size of the target itself. Objects with larger physical dimensions typically generate longer radar target clouds, while smaller objects produce shorter clouds.

**Figure 7:**
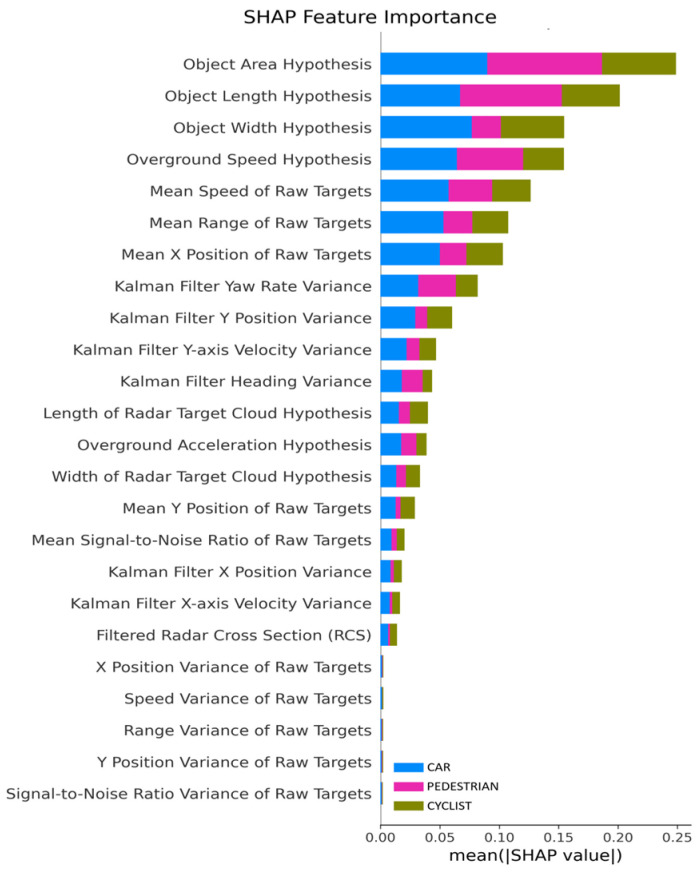
SHAP Feature Importance.

An additional analysis was conducted to support the findings obtained from the Pearson correlation heatmap. For this purpose, SHAP (SHapley Additive exPlanations) analysis was implemented to provide a deeper understanding of feature importance and individual feature contributions to the model’s predictions, the power of SHAP being described in ref. [24]. The SHAP summary plot visualizes the impact of each feature on the output of the model across the dataset. Figure 7 illustrates the SHAP feature importance analysis, highlighting the relative contribution of each input variable to the model’s classification performance across object classes such as Car, Pedestrian, and Cyclist. The most influential feature identified is the Object Area Hypothesis, which demonstrates a strong discriminative capability due to the inherent differences in object dimensions across classes. This feature effectively captures the spatial footprint of objects, making it a robust indicator for class separation. The second most important feature is the Object Length Hypothesis, which further enhances classification accuracy by leveraging the longitudinal dimension of objects, a characteristic that varies significantly between categories such as pedestrians and vehicles. Although the Object Width Hypothesis ranks third in importance, it is considered redundant due to its derivation from the same geometric components that define object area. Consequently, it may be excluded from the final feature set to reduce dimensionality without compromising model performance.

In the final feature selection process for radar-based object classification, it is essential to consider physical characteristics such as Signal-to-Noise Ratio (SNR) and Radar Cross Section (RCS), which significantly contribute to the generalization and robustness of machine learning models. While kinematic features, such as speed, acceleration, describe dynamic behavior that can vary widely across scenarios, SNR and RCS provide intrinsic, relatively stable information about the object’s detectability and reflective properties. SNR reflects the clarity of the radar return relative to background noise, indicating how reliably an object can be distinguished, whereas RCS quantifies the reflected radar energy, influenced by the object’s shape, size, orientation, and material composition. The inclusion of RCS in classification tasks has demonstrated considerable efficacy in automotive radar applications. For instance, Nebhwani and Dashpute [25] showed that temporal RCS patterns extracted from 77 GHz radar enabled real-time classification between ground and non-ground objects, supporting robust feature extraction. Tatarchenko and Rambach [26] further advanced this approach by employing histogram-based RCS features within a deep learning model, achieving high classification accuracy and robustness against noise and missing data. Complementing these findings, Coşkun and Bilicz [27] demonstrated that RCS histograms could be effectively used to cluster and classify 14 different vehicle types in real-world driving conditions, highlighting the discriminative power of RCS-based features in complex and variable environments.

### 3.4. Neural Networks Architecture Based on Grid Search

Here we extend the method presented in ref. [9]. In this study, we employed the same feature set and neural network architecture as presented in our previous work; however, we utilized an updated dataset and computed additional evaluation metrics to gain deeper insight into the model’s performance.

In the design of the neural network model, the initial step involved determining the optimal set of hyperparameters through a grid search approach. The hyperparameters considered included batch size, number of epochs, dropout rate, optimizer type, learning rate, and the number of hidden units. Based on the results of this search, the selected configuration comprised a batch size of 64, a dropout rate of 0.1, 50 training epochs, 10 neurons per hidden layer, a learning rate of 0.01, and the RMSprop optimizer. RMSprop was chosen due to its computational efficiency and its suitability for datasets with limited variation, making it an ideal choice for the current classification task.

The neural network architecture is the same in ref. [9], consisting of a flattened input layer, two hidden dense layers, and a dense output layer. Each hidden layer contained 10 neurons and utilized the ReLU activation function to introduce non-linearity. The output layer, designed for a three-class classification problem, employed the SoftMax activation function to produce a probability distribution over the classes. The loss function used was sparse categorical crossentropy, which is appropriate for multi-class classification tasks involving integer-labeled targets. The dataset was partitioned into training, validation, and testing subsets in a 60%:20%:20% ratio, respectively.

Unlike the previous approach, where training was halted upon reaching an accuracy threshold of 99%, this iteration excluded such a constraint due to the increased dataset size. Instead, early stopping was implemented to mitigate overfitting, with a patience parameter of 5 and monitoring based on validation accuracy. Additionally, the ReduceLROnPlateau callback was employed to dynamically adjust the learning rate during training. This mechanism reduced the learning rate by a factor of 0.1 when the validation accuracy plateaued for three consecutive epochs, thereby preventing the model from overshooting optimal solutions and promoting more stable convergence.

**Figure 8:**
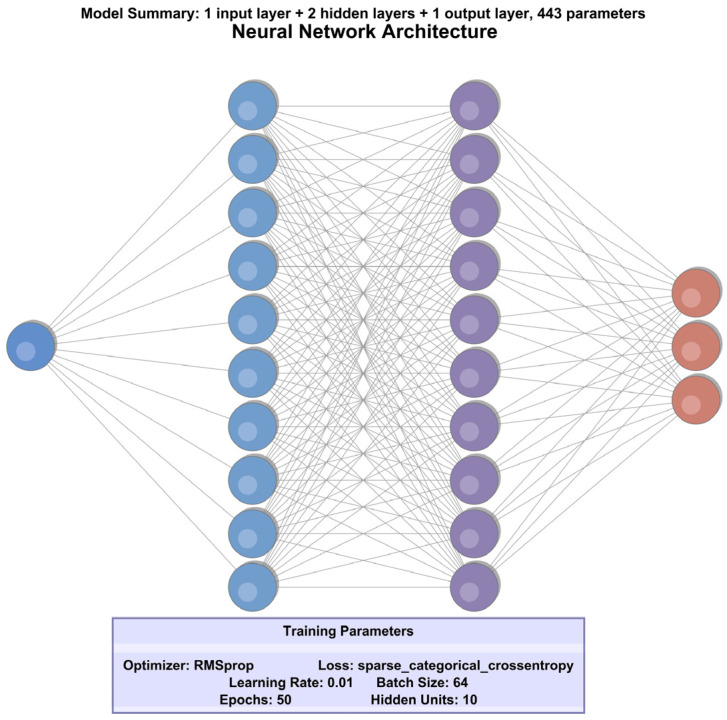
Neural Network Architecture. The hyperparameters with the best results are batch size: 64; epochs: 50; optimizer: RMSprop; learning rate: 0.01; number of neurons: 10; number of hidden layers: 2.

Figure 8 shows the architecture used for the neural network for the first method proposed.

In our study, we address the issue of class imbalance in a multi-class classification task. The dataset used exhibits a significant imbalance among these classes, with the “car” class being overrepresented compared to the “pedestrian” and “cyclist” classes. This imbalance can severely affect the performance of the model, as it tends to bias predictions toward the majority class, leading to poor generalization and reduced accuracy for the minority classes. To mitigate this issue, we employed the Synthetic Minority Over-sampling Technique (SMOTE) [8], a widely recognized method for generating synthetic samples of the minority classes. To ensure reproducibility across different runs, a fixed random seed was set. SMOTE works by creating new synthetic instances of the minority classes through interpolation between existing samples and their nearest neighbors in the feature space. This approach increases the representation of underrepresented classes without simply duplicating existing data, thereby enhancing the diversity and informativeness of the training set. In our context, applying SMOTE ensures that the neural network receives a more balanced and representative view of all three object categories during training. This not only improves the model’s ability to correctly classify pedestrians and cyclists but also contributes to a more robust and fair classification system overall. The use of SMOTE is thus a critical step in enhancing the performance and reliability of our neural network in real-world scenarios where class distributions are inherently uneven.

### 3.5. Neural Network Architecture Based on Genetic Algorithm

In the design process of the neural network for the second method, we employed a Genetic Algorithm to achieve rapid convergence to an optimal solution. A genetic algorithm is a heuristic search inspired by the process of natural selection, where potential solutions evolve over generations through selection, crossover, and mutation. This method is particularly effective for optimizing complex problems with large search spaces.

In the construction of architecture for neural networks, we consider as hyperparameters the following: batch size, epochs, optimizer (Adam, Root Mean Square Propagation), learning rate, number of neurons in the hidden unit and the number of layers.

For the genetic algorithm, we considered the following parameters: population size of 40, number of generations set to 10, crossover probability of 0.4, and mutation probability of 0.0. These parameters were chosen to enhance genetic diversity, ensure thorough exploration of the search space, and promote the exchange of beneficial traits. These parameters were selected heuristically, guided by values commonly reported in related studies and validated through preliminary experimentation. Such moderate population sizes, low mutation rates, and limited generations have been shown to achieve robust convergence with manageable computational costs in feature selection and classification tasks [28,29,30,31]. These settings balance search effectiveness with efficiency, avoiding excessive runtime while maintaining solution quality. Parallelization was implemented using Python’s DEAP framework [32] in combination with the multiprocessing module, replacing DEAP’s default sequential mapping with a parallel map function to evaluate all individuals in a generation concurrently. This embarrassingly parallel approach, well-suited to independent fitness evaluations, enables near-linear speedup as the number of available CPU cores increases.

The parallel GA workflow distributes fitness evaluations across a fixed pool of worker processes, synchronizing results at the end of each generation before applying genetic operators. This method has demonstrated substantial runtime reductions in hyperparameter optimization and feature selection without degrading accuracy [33,34,35]. The efficiency gains stem from the independence of individual evaluations, allowing effective utilization of multi-core architectures. Upon completion, the multiprocessing pool is closed and joined to ensure proper resource management. Such parallelization strategies not only accelerate the evolutionary search process but also improve scalability, making them particularly advantageous for computationally intensive wrapper-based feature selection problems. The first step we need to take in the neural network is to ensure that all the data has the same range. For this, we will apply a MinMaxScaler, ensuring uniform feature contribution, optimizing gradient descent efficiency, and reducing computational complexity. Initially, we applied the MinMaxScaler to the training subset of the dataset. Subsequently, the learned scaling parameters were utilized to transform the validation subset, ensuring consistency in feature scaling across both training and validation data.

We continue to use the Synthetic Minority Over-sampling Technique (SMOTE) [8] to address the imbalance in the dataset, to generate synthetic data for underrepresented classes. The following two tables present a quantitative summary of the synthetic data generated. Specifically, they detail the number of artificially generated samples per class and the proportion of synthetic samples relative to the original dataset.

Table 2 presents the number of samples per class in the dataset prior to the application of the SMOTE. The Car class dominates the dataset, with a total of 830,764 samples, accounting for approximately 79.2% of the overall data. In contrast, the Pedestrian and Cyclist classes are significantly underrepresented, comprising only 13.7% and 7.0% of the dataset, respectively. Table 3 reports the number of synthetic samples generated for each minority class following the application of the SMOTE. The objective was to balance the dataset by increasing the size of each minority class (Pedestrian and Cyclist) to match that of the majority class (Car), which contained 830,764 samples. As shown, SMOTE generated 686,730 synthetic samples for the Pedestrian class (47.6%) and 756,987 for the Cyclist class (52.4%). The Car class did not require any synthetic data generation and is therefore not included in this table. This oversampling process effectively mitigated the class imbalance, creating a more uniform distribution across all classes.

To gain a deeper understanding of the characteristics of the synthetic data generated by SMOTE, a comparative feature distribution analysis was conducted. This analysis examined and contrasted the distributions of key features across the original and synthetic datasets. By evaluating the alignment between these distributions, we aimed to assess the consistency and quality of the artificially generated samples and their ability to preserve the statistical properties of the original data.

Table 4 provides a statistical comparison between the original and synthetic datasets for two object classes: pedestrian and cyclist. The evaluation includes key statistical measures: mean, standard deviation (Std), and the Kolmogorov–Smirnov (KS) test, to assess how well the synthetic data generated by SMOTE replicates the distributional properties of the original data. For both pedestrian and cyclist classes, the mean differences across features between original and SMOTE-generated synthetic data are consistently small (0.0006–0.02), indicating that the synthetic data effectively preserves the central tendency of the original distributions. Key discriminative features, such as Filtered Radar Cross Section (RCS), Overground Speed Hypothesis, and Object Length Hypothesis, exhibit particularly low deviations (e.g., 0.0012 for pedestrian RCS, 0.0007 for cyclist object length), reflecting high fidelity in replicating representative characteristics. Variability is also well maintained, with standard deviation ratios, defined as Std(Synthetic)/Std(Original), generally ranging from 0.92 to 1.00, although slight underestimations (e.g., 0.9285 for pedestrian object length, 0.9552 for cyclist cloud width) suggest moderate smoothing from SMOTE’s interpolation process. Kolmogorov–Smirnov (KS) statistics further confirm distributional similarity, with most features showing low KS values (<0.05) and non-significant *p*-values (e.g., pedestrian RCS *p* = 0.991, cyclist RCS *p* = 0.987). However, features such as Hypothesized Area of Object for both classes yield extremely low *p*-values (e.g., 1.02 × 10^−104^ for pedestrian, 1.37 × 10^−117^ for cyclist), indicating distributional differences despite minimal mean deviations.

The statistical comparison demonstrates that the SMOTE-generated synthetic data effectively preserves the key distributional properties of the original data for both pedestrian and cyclist classes. The synthetic samples replicate the original data’s central tendency and spread with high fidelity in most features. Although slight deviations in distributional shape exist, as indicated by some low KS *p*-values, these are not widespread and do not appear to compromise the representativeness of the synthetic data. Consequently, the SMOTE appears to have generated high-quality synthetic samples, suitable for mitigating class imbalance while preserving the integrity of the original feature space.

To evaluate the quality and distribution of the synthetic data generated for model training, a two-step t-distributed Stochastic Neighbor Embedding (t-SNE) analysis was conducted. The goal was to assess whether the synthetic samples accurately represent the underlying data manifold without introducing aberrant or unrealistic values.

**Figure 9:**
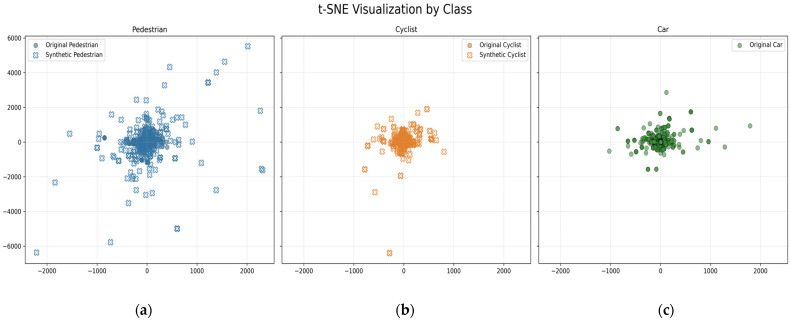
t-SNE results: (**a**) visualization of pedestrian (original and synthetic data), (**b**) visualization of cyclist (original and synthetic data), (**c**) visualization of car (original data only).

Figure 9 presents the t-SNE visualization of the original and SMOTE-generated synthetic samples for each object class. For the pedestrian case, see Figure 9a, the synthetic samples (blue crosses) closely overlap with the original samples (blue dots), indicating that SMOTE successfully replicates the underlying distribution and spatial variability of this minority class. Similarly, for the cyclist class (Figure 9b), synthetic samples (orange crosses) are well integrated within the distribution of original samples (orange dots), demonstrating effective augmentation and preservation of the class’s feature space structure. In contrast, for the car class (Figure 9c), only original samples (green dots) are shown, as SMOTE was not applied to this majority class to avoid introducing class imbalance or overrepresentation. The visual consistency between synthetic and original samples for the minority classes supports the quality of the data augmentation process and its potential to enhance classifier performance without distorting the intrinsic feature relationships. To enhance the robustness of the model, we employed a dropout technique, specifically discarding one neuron from each hidden layer. This approach is crucial for improving the final convergence of the model. Dropout helps prevent overfitting by randomly omitting neurons during training, thereby forcing the network to learn more generalized patterns.

As a loss function for our neural network, we decided to use sparse categorical cross-entropy due to its memory efficiency and well-behaved gradients, which are essential for effective backpropagation in multi-class classification problems with integer class labels. This loss function computes the cross-entropy between the predicted probability distribution and the actual distribution, represented by the integer class label. Specifically, it evaluates the difference between the predicted probabilities for each class and the actual class label, focusing only on the probability assigned to the true class. This ensures that the loss reflects how well the model’s prediction aligns with the true class, with only the term corresponding to the true class contributing to the loss. By doing so, it effectively minimizes the error associated with the correct classification, thereby enhancing the model’s accuracy and performance.

This choice, combined with adaptive training strategies such as early stopping and learning rate modulation, enhances generalization by preventing overfitting and optimizing computational resources. Early stopping halts training when performance on unseen data begins to deteriorate, while learning rate modulation helps navigate the optimization landscape by reducing the learning rate when the monitored metric plateaus, thereby ensuring robust classification performance.

**Figure 10:**
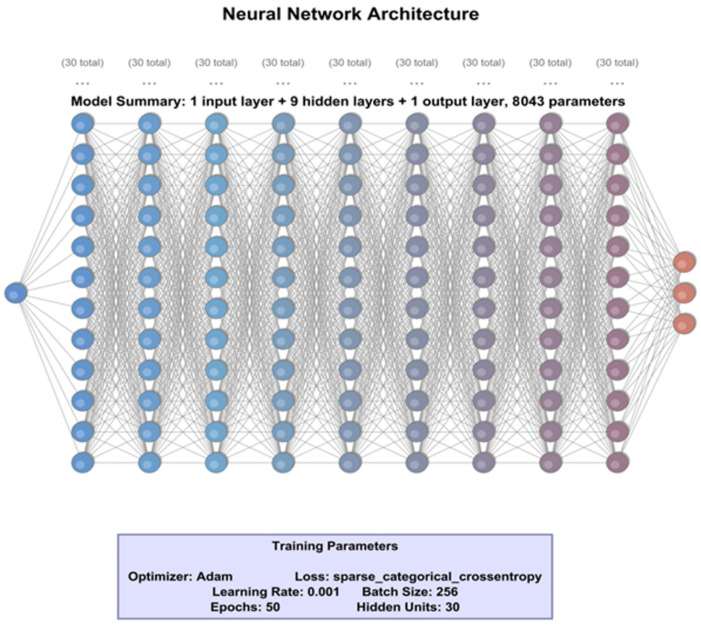
Neural Network Architecture. The hyperparameters with the best results are batch size: 256; epochs: 50; optimizer: Adam; learning rate: 0.001; number of neurons: 30; number of hidden layers: 9.

The neural network architecture presented in Figure 10 represents the final configuration selected for our classification task and is the result of applying a genetic algorithm to optimize the hyperparameter space. Unlike the Grid Search-based method, where a smaller neural network was used, the current approach uses a more complex model, allowing for a more rigorous evaluation of the network’s capabilities.

The genetic algorithm employed in this work is a population-based metaheuristic inspired by the principles of natural selection and evolution. It iteratively evolves a population of candidate solutions, each representing a unique combination of hyperparameters such as batch size, number of epochs, optimizer type, learning rate, number of neurons, and number of hidden layers, through operations like selection, crossover, and mutation. By evaluating the fitness of each candidate based on model performance metrics (e.g., accuracy, precision, recall), the algorithm converges toward an optimal or near-optimal configuration. This approach is particularly advantageous in high-dimensional search spaces, where exhaustive methods like grid search become computationally prohibitive. In our implementation, the genetic algorithm was executed in parallel to accelerate convergence, ultimately yielding a neural network architecture that balances complexity and performance effectively.

We maintain a consistent data partitioning strategy to ensure robust model training and evaluation. Specifically, the dataset is divided into three subsets: 60% is allocated for training, 20% for validation, and the remaining 20% for testing. This ratio is chosen to provide a sufficient amount of data for the model to learn effectively, while also preserving distinct subsets for tuning hyperparameters and assessing generalization performance.

To assess the risk of over-parameterization, the ratio between the number of training samples and model parameters was calculated. The model has 8043 trainable parameters and was trained on approximately 1,495,375 samples (this includes the real data and synthetic data), resulting in a data-to-parameter ratio of approximately 186:1. This ratio suggests that the model has sufficient data to generalize effectively without overfitting, assuming proper regularization and training procedures are applied.

## 4. Results

In the following, we present the results obtained from two distinct neural network optimization strategies: one based on grid search and the other on a genetic algorithm. Both approaches were applied to the same classification task involving three object categories: car, pedestrian, and cyclist. Accuracy, precision, recall, and F1 score were computed for each class. For the overall performance model’s performance, micro-averaged metrics were also calculated. These are practically the same, due to applying the SMOTE procedure. A commercial millimeter-wave automotive radar was employed to evaluate the proposed algorithm. The device features high resolution in distance, relative velocity, and angle measurements, operates at a center frequency of 76.5 GHz, provides azimuth and elevation angle fields of view of ±90° and ±15°, respectively, and utilizes an Ethernet communication interface.

### 4.1. Results for Neural Network Based on Grid Search

In this section we show results of the neural network model, where hyperparameter optimization was conducted using grid search.

**Figure 11:**
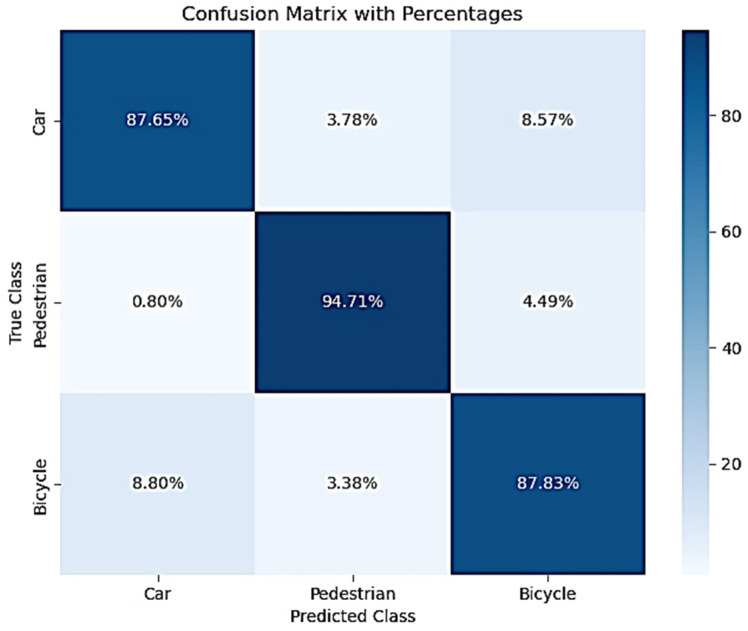
Confusion Matrix—based on grid search (Validation Dataset).

**Figure 12:**
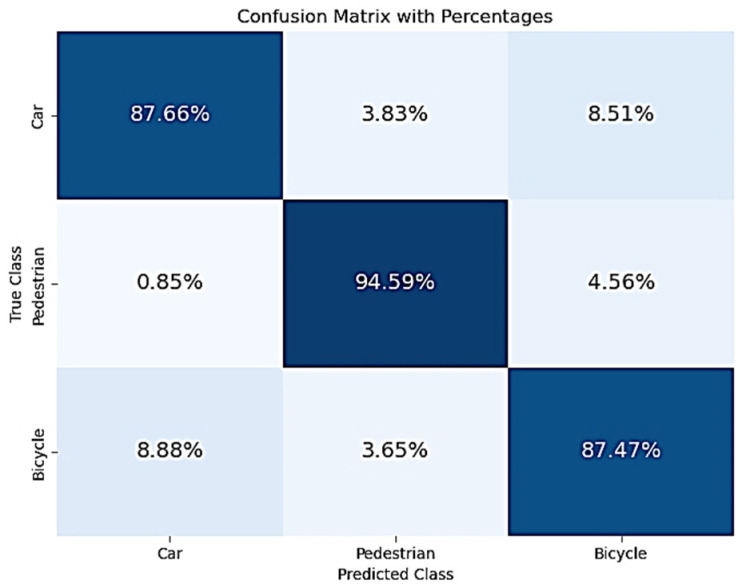
Confusion Matrix—based on grid search (Test Dataset).

The classification performance of Method 1 (grid search) is presented in Table 5 and Table 6, with corresponding confusion matrices in Figure 11 and Figure 12. Despite its lightweight architecture, the model achieved strong and balanced performance across all classes, with F1-score of around 90% for both datasets. The marginal drop between validation and test F1 indicates robust generalization, validating the effectiveness of the grid search procedure in identifying well-performing hyperparameters and SMOTE procedure.

Concerning class-specific performance, the pedestrian class achieved the highest accuracy on both validation and test phases, with high precision and F1-score. The confusion matrices in both phases show low misclassification rates. The cyclist class, typically challenging in classification, has the highest F1-score overall on both phases. The recall exceeds precision, suggesting the model is effective at detecting cyclists with few missed instances. However, confusion matrices show that cyclists are sometimes misclassified as cars, which may reflect similarities in radar signatures at certain speeds or angles. Notable challenge arises when an object enters the radar’s field of view near its boundary. In such cases, the radar system typically receives only a limited number of reflections from the object, often capturing data from a small portion of its surface. This partial observation hinders the system’s ability to accurately characterize the object’s full geometry and motion, thereby increasing the likelihood of misclassification. The car class showed the lowest F1-score, primarily due to confusion with cyclists and less with pedestrians. This can be explained by the small search space used for the grid search. However, precision and recall are balanced, indicating that misclassification is not skewed toward false positives or negatives. The confusion matrices show that the car class is the most confused, particularly with cyclists. This misclassification remains consistent across validation and test datasets, suggesting a systemic overlap rather than data noise. Pedestrian classification is highly reliable, with over 94% accuracy and low cross-class misidentification, which is crucial in safety-critical applications. Cyclist classification, while overall strong, shares some confusion with both cars and pedestrians, suggesting that minority classes may still benefit from further feature disentanglement.

The SMOTE use improved classification performance for the pedestrian and cyclist classes, which are typically underrepresented in automotive radar datasets. The high recall values for these classes indicate that SMOTE successfully addressed class imbalance without harming the generalization ability of the model. As such, method 1 demonstrates that even a compact neural network architecture, when properly tuned via grid search and supported by class-balancing techniques like SMOTE, can achieve high-performance, generalizable object classification in automotive radar applications. The model’s ability to accurately detect vulnerable road users (pedestrians and cyclists), combined with its simplicity, makes it particularly suitable for embedded systems and real-time processing in autonomous driving or ADAS platforms.

### 4.2. Results for Neural Network Based on Genetic Algorithm

**Figure 13:**
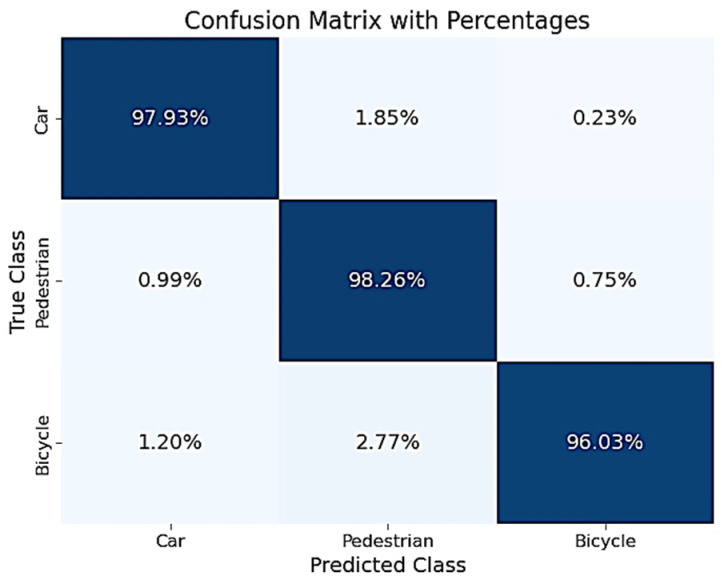
Confusion Matrix—based on the genetic algorithm (Validation Dataset).

**Figure 14:**
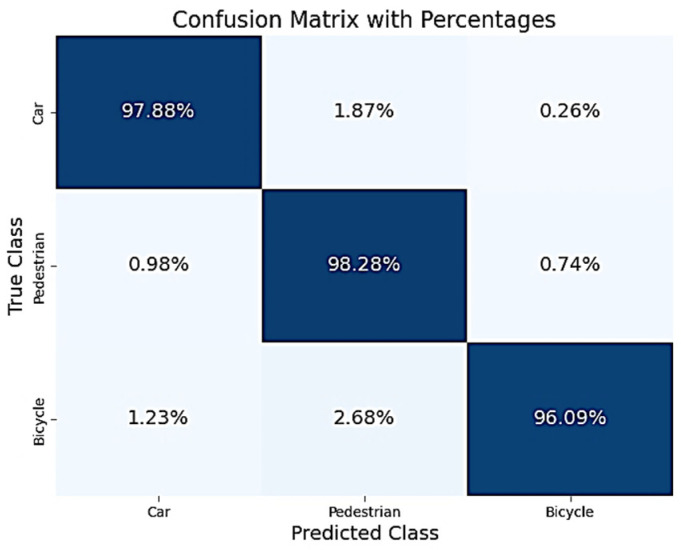
Confusion Matrix—based on the genetic algorithm (Test Dataset).

In this section we show results of the neural network model, where hyperparameter optimization was conducted using a genetic algorithm. The validation results of the neural network, obtained using hyperparameters optimized through a genetic algorithm (Method 2), indicate a high level of predictive consistency across all evaluated classes. Table 7 and Table 8, along with Figure 13 and Figure 14, present the performance of the neural network trained using Method 2, which employs a genetic algorithm for hyperparameter optimization. The model achieved very good results on both the validation and test datasets, far exceeding the Method 1 performance. The consistently high F1 scores across datasets indicate strong generalization capability, suggesting that the model avoids overfitting despite its depth. This method shows a notable performance improvement over Method 1 across all metrics and datasets.

Performance on the test dataset mirrors the validation set almost exactly, suggesting excellent generalization and low risk of overfitting, despite the model’s complexity. These results highlight the effectiveness of genetic algorithms in identifying high-performing hyperparameter configurations, especially for deeper networks.

The pedestrian class achieved an F1-score of 96.9% on the test dataset, up from 88.82% in Method 1, while the recall of 98.28% reflects the model’s sensitivity to vulnerable road users, which is crucial for safety-critical applications. The confusion with cyclists is minimal (≤0.75%), and with cars, even lower (<1%). The cyclist class performance has improved significantly: F1-score jumped from ~93.6% to 97.5% with a high precision (98.97%) and low false positives, suggesting that the model confidently distinguishes cyclists, a historically challenging class. The largest misclassification (2.68%) still occurs with pedestrians, but this is reduced compared to Method 1. The car class classification results show an accuracy increased to 97.88%, with very few misclassifications (<2%), a strong result considering this class’s prior confusion in Method 1. Precision and recall are nearly perfectly balanced (~97.8%), indicating strong model calibration.

Analyzing the confusion matrices in Figure 13 and Figure 14, the majority of predictions are correctly classified, with minimal confusion between classes, and we see that misclassification rates across all classes are significantly reduced compared to Method 1. The model has true positive rates near or above 96% for all classes. The remaining confusion is minimal and largely symmetrical between cyclists and pedestrians, which is likely due to shared spatial features or radar signatures at certain orientations.

Table 9 presents various combinations of hyperparameters and their corresponding performance metrics, providing a clearer understanding of how different hyperparameter configurations influence the performance of the neural network. The optimization parameter is encoded such that a value of 0 corresponds to the Adam optimizer, while a value of 1 corresponds to the RMSprop optimizer. The reported values for Precision, Recall, and F1-score represent the mean values across all classes. The best classification performance was achieved using the parameter configuration reported in Table 9, namely a batch size of 256, 50 training epochs, the Adam optimizer, a learning rate of 0.001, 30 hidden units, and 9 layers, which was subsequently used in Method 2.

The proposed method is particularly effective in urban environments, where the movement patterns of target objects are relatively uniform and can be accurately characterized using features such as absolute velocity, acceleration, and other derivatives describing the object’s kinematics. By leveraging a genetic algorithm, the system not only learns these motion patterns more effectively but also enhances geometric characterization by directly measuring the raw radar target cloud’s width and length. Furthermore, it integrates features that are independent of kinematics, such as Radar Cross Section (RCS) and Signal-to-Noise Ratio (SNR). In radar sensing, RCS serves as a robust indicator of an object’s reflective properties, influenced by its size, shape, and material composition, while SNR reflects the clarity and detectability of the target amidst noise, both offering reliable signatures for object identification regardless of movement. This combination of kinematic, geometric, and independent radar descriptors enables a richer and more discriminative feature set, ultimately improving classification accuracy and robustness in dense, complex urban scenarios.

While the method’s performance in urban contexts is compelling, achieving similar generalization across a broad spectrum of driving environments, such as highways or extra-urban settings, presents challenges. This is primarily due to the limited applicability of kinematic descriptors when target motion patterns vary drastically between scenarios. Building a sufficiently diverse dataset to capture this variability can be resource-intensive, highlighting the trade-off between scenario-specific optimization and universal generalization.

These findings validate the effectiveness of the genetic algorithm in identifying a hyperparameter configuration that supports robust and generalizable model performance during testing.

## 5. Discussion

In this section, we present a comparative analysis of the results obtained from our two models.

### 5.1. Model Architecture and Hyperparameter Optimization

Method 1 produced a lightweight architecture through grid search, consisting of two hidden layers with 10 neurons each, optimized with RMSprop, a batch size of 64, and a learning rate of 0.01. In contrast, Method 2, through genetic search, converged on a deeper architecture with nine hidden layers, each containing 30 neurons, trained with the Adam optimizer, a larger batch size of 256, and a lower learning rate of 0.001.

The significant difference in architecture reflects the nature of the two optimization strategies. Grid search exhaustively evaluates combinations in a fixed hyperparameter space, often leading to simpler, robust solutions. Genetic algorithms, by contrast, explore a broader and more complex search space through evolutionary operations, capable of discovering deeper, more expressive architectures. While Method 2’s deeper model has greater representational capacity, it comes at the cost of increased training complexity and resource demands. The hyperparameter optimization for Method 1 completed in approximately 12 h, while Method 2 required three days, emphasizing the computational burden associated with evolutionary methods. In Section 5.4, runtime measurements were conducted on prediction of the neural network architectures obtained through the two hyperparameter optimization methods: grid search and genetic algorithm.

### 5.2. Feature Utilization and Dimensionality Considerations

Interestingly, Method 1 uses 29 input features, whereas Method 2 achieved superior performance using only 16 features. Feature reduction in Method 2 likely contributed to its higher generalization performance and faster inference, despite the deeper architecture. Lower-dimensional input spaces reduce overfitting risks and computation per forward pass, which is particularly advantageous in real-time automotive applications. It also significantly decreases computational time, enabling the exploration of a larger hyperparameter search space and the use of more extensive datasets.

### 5.3. Classification Performance

Across all evaluated metrics, Method 2 outperforms Method 1 by a significant margin. On the test dataset:

- F1-score improved from 89.91% (Method 1) to 97.41% (Method 2).
- Recall for pedestrian and cyclist classes, which are critical for safety, rose from ~88–94% to 98.28% and 96.09%, respectively.
- Confusion matrices show a marked reduction in misclassifications, particularly for cyclist-pedestrian overlap, known to be challenging due to similar radar signatures.

The confusion matrix for Method 1 revealed persistent misclassification of cyclists as cars (8.57%) and pedestrians (3.38%). In contrast, Method 2 achieved cyclist misclassification rates below 3%, suggesting improved feature discrimination and robustness.

The minimal performance degradation observed between the validation and test datasets for both classification methods indicates a high degree of generalization. This is particularly noteworthy given the substantial increase in dataset size compared to prior work [9], which introduces greater variability and potential for overfitting. The consistency of performance metrics across datasets indicates that both models have effectively learned generalizable patterns rather than memorizing training data.

The robustness of Method 2 can be attributed to several architectural and methodological factors. First, the use of a genetic algorithm enabled exploration of a broader and more expressive hyperparameter space, allowing the model to converge on an architecture that balances complexity with generalization. Second, the application of dropout regularization and early stopping during training mitigated overfitting by preventing the model from becoming overly reliant on specific neurons or training patterns. Third, the use of SMOTE to address class imbalance ensured that minority classes were adequately represented during training, enhancing the model’s ability to generalize across all object categories.

In contrast, while Method 1 also exhibited strong generalization, the slightly larger performance gap between validation and test datasets (~0.15%) may reflect its more constrained architecture and limited hyperparameter search space. In the end, the model’s ability to maintain high accuracy and F1-scores across datasets confirms its reliability as a lightweight alternative for real-time applications.

### 5.4. Computational and Deployment Considerations

With respect to the computational cost and deployment feasibility, Method 1 employs a lightweight architecture with two hidden layers and 10 neurons per layer, resulting in a compact model with low memory requirements and minimal inference latency. This makes it particularly well-suited for deployment on embedded systems such as automotive electronic control units (ECUs) or edge AI accelerators. On the other hand, Method 2 utilizes a deeper architecture with nine hidden layers and 30 neurons per layer, which, while achieving higher classification accuracy, incurs greater computational and memory demands. This model is more appropriate for deployment on centralized processing units or cloud-based platforms where computational resources are abundant.

To assess the suitability of the proposed neural network architectures for deployment in resource-constrained environments such as embedded automotive systems, runtime performance and memory footprint were evaluated on a workstation equipped with an Intel i7-1165G7 CPU and 32 GB RAM. Table 10 reports key metrics, including inference time, latency, throughput, memory usage, floating point operations (FLOPs), and parameter count. 

The grid search–optimized model comprises 443 parameters with a memory footprint of 0.00196 MB, while the genetic algorithm-optimized model contains 8043 parameters and requires 0.03281 MB. Correspondingly, FLOPs are substantially higher for the genetic model (15,828 vs. 878), reflecting its increased architectural complexity. Despite its larger size, the genetic model achieved faster inference for batch size 1 (53.55 ms vs. 59.13 ms), likely attributable to its reduced input dimensionality (16 vs. 29 features). At batch size 256, both models exhibited low latency (0.23 ms/sample), with the genetic model demonstrating marginally higher throughput (4389.25 vs. 4297.19 samples/s). These results underscore the efficiency gains achievable through feature dimensionality reduction, even in deeper architectures, and indicate that both models are viable for real-time automotive applications, particularly when deployed on high-performance electronic control units (ECUs) or dedicated inference accelerators. This aligns with findings from recent studies [36,37], which report improved prediction accuracy and computational efficiency through deep learning and evolutionary optimization in dynamic, real-time environments.

## 6. Conclusions

We considered the problem of object classification in automotive radar systems, with three classes: car, pedestrian, and cyclist. We proposed two neural network-based architectures, using two different hyperparameter search techniques: grid search and the genetic algorithm. The grid search-based model (Method 1) has a simpler architecture with two hidden layers with 10 neurons each, optimized with RMSprop, a batch size of 64, and a learning rate of 0.01. The genetic algorithm model (Method 2) has a deeper architecture with 9 hidden layers and 30 neurons per layer, trained with the Adam optimizer, a larger batch size of 256, and a learning rate of 0.001. Model 1 uses 29 input features, while Model 2 achieved superior performance using only 16 features. The attributes used for classification are kinematic correlations and motion for the first method and kinematic motion for the second one, object dimensions for both methods and signal-to-noise ratio (SNR), radar cross-section (RCS) and Kalman filter-based motion descriptors for the second one. The grid search model accuracy is 90.06% on the validation dataset and 90.00% on the test dataset. The genetic algorithm model, which leverages a more complex feature set and achieves better results, has an overall accuracy of about 97.40% on both validation and test datasets.

Our study demonstrates the efficacy of neural network-based radar object classification in urban automotive environments using two different techniques for hyperparameter search. The results demonstrate that Method 2 significantly outperforms Method 1, for example, Method 2 exhibited higher precision and recall for critical classes such as pedestrians and cyclists, essential for the safety and reliability of autonomous driving systems. Despite this, Method 2 also comes with a higher computational cost during training and a much deeper architecture (9 layers vs. 2 in Method 1). This model was able to achieve a very good performance using a reduced feature set, highlighting the effectiveness of the genetic algorithm in neural architecture optimization. In contrast, Method 1 offers a more computationally efficient solution with lower training time and simpler architecture, which means it could be better suited for resource-constrained or real-time embedded systems. This comes at reduced accuracy, for example, for the class car.

The challenges of manual labeling in dense urban scenarios highlight the need for further refinement and the development of automated labeling techniques. Future research should focus on enhancing the robustness and generalization capabilities of the model, potentially through the integration of additional features, features that are independent from the kinematics of the object, which can improve the generalization of classification; being independent of the scenario; and more diverse training data. This work contributes to the advancement of reliable and accurate radar-based object classification systems, which are crucial for the safe and efficient operation of autonomous vehicles in complex urban environments. In addition, the proposed classification framework can be extended by integrating data from multiple radar units with camera systems, forming a multi-sensor fusion platform, leveraging the complementary strengths of radar and vision. Radar provides reliable spatial and velocity information under diverse environmental conditions, while cameras contribute rich semantic and contextual data. Such fusion could offer enhanced spatial and temporal resolution, improving object discrimination and robustness in complex traffic environments. Future work could focus on implementing this fusion strategy to improve generalization across varied traffic scenarios and enable real-time deployment on embedded systems, such as those used in autonomous vehicles or advanced driver-assistance systems (ADAS).

## Figures and Tables

**Table 1:** Feature ordered by rank (last column). A feature is more important if its mean has a higher value and, respectively, its rank has a lower value.

Feature	1–3	1–5	3–5	Mean	Rank
rcs	71.7637	150	78.2363	100	6
length	50.9817	140.684	89.7022	93.7892	8
classHighest	72.6151	120.86	48.2448	80.5733	9
xyVelAbs	19.5813	65.8411	46.2598	43.894	20
stdDevYawRate	25.8782	51.8747	25.9965	34.5832	21
fusionInfo	30.4214	42.1661	11.7446	28.1107	31
corrYPosOrientation	8.074	29.086	37.16	24.7733	44
width	5.17907	142.046	136.867	94.6975	46
corrXPosOrientation	25.1651	5.78532	19.3798	16.7767	50
corrXPosVelocityAbs	24.0552	16.8064	7.24877	16.0836	54
existenceProbability	24.8604	29.9334	5.07303	19.9556	56

**Table 2:** Number of data points for each class before SMOTE.

Class	Number of Samples	Percentage
Pedestrian	144,034	13.7%
Cyclist	73,777	7.0%
Car	830,764	79.2%

**Table 3:** Number of artificially generated data points for each class after SMOTE.

Class	Number of Samples	Percentage
Pedestrian	686,730	47.6%
Cyclist	756,987	52.4%

**Table 4:** Statistics on original and synthetic data.

Class	Feature	Mean (Orig.)	Mean (Synth.)	Mean Diff.	Std (Orig.)	Std (Synth.)	Std Ratio	Ks Statistic	Ks *p*-Value
Pedestrian	Length of RTC Hypothesis	0.2727	0.2695	0.0032	0.3029	0.2862	0.9448	0.0148	5.48 × 10^−23^
Cyclist		1.0580	1.0567	0.0014	0.9291	0.9088	0.9782	0.0158	5.93 × 10^−15^
Pedestrian	Overground Speed Hypothesis	1.6249	1.6162	0.0087	1.4116	1.3834	0.9800	0.0083	1.56 × 10^−7^
Cyclist		4.3023	4.2835	0.0188	2.4761	2.4318	0.9821	0.0081	3.05 × 10^−4^
Pedestrian	SNR Variance of Raw Targets	18.5336	18.5206	0.0130	44.6048	44.6857	1.0018	0.0033	0.153
Cyclist		32.3015	32.3050	0.0035	65.5925	65.5351	0.9991	0.0056	0.0305
Pedestrian	Filtered RCS	−0.3827	−0.3839	0.0012	5.2821	5.2458	0.9931	0.0013	0.991
Cyclist		−0.0435	−0.0455	0.0021	7.0644	7.0316	0.9954	0.0017	0.9869
Pedestrian	Width of RTC Hypothesis	0.0541	0.0535	0.0006	0.1000	0.0958	0.9585	0.0222	1.86 × 10^−51^
Cyclist		0.1322	0.1311	0.0012	0.1760	0.1681	0.9552	0.0204	7.87 × 10^−25^
Pedestrian	Overground Acceleration Hypothesis	0.3682	0.3626	0.0056	0.7260	0.7131	0.9822	0.0183	3.16 × 10^−35^
Cyclist		0.8365	0.8244	0.0120	1.1133	1.0814	0.9713	0.0251	3.63 × 10^−37^
Pedestrian	Object Length Hypothesis	0.6372	0.6317	0.0055	0.2988	0.2774	0.9285	0.0318	1.02 × 10^−104^
Cyclist		2.1337	2.1330	0.0007	0.7311	0.7144	0.9772	0.0426	1.19 × 10^−106^
Pedestrian	Mean SNR of Raw Targets	17.6271	17.6197	0.0074	9.2725	9.2639	0.9991	0.0011	0.9981
Cyclist		20.1203	20.1124	0.0079	11.0068	10.9789	0.9975	0.0016	0.9936
Pedestrian	Hypothesized Area of Object	0.3261	0.3232	0.0030	0.1656	0.1524	0.9199	0.0318	1.02 × 10^−104^
Cyclist		1.4655	1.4587	0.0068	1.3991	1.3815	0.9874	0.0448	1.37 × 10^−117^

**Table 5:** Neural Network Results—based on grid search (Validation Dataset).

Class	Accuracy (%)	Precision (%)	Recall (%)	F1 Score (%)
car	87.65%	87.06%	87.83%	87.44%
pedestrian	94.71%	90.13%	87.65%	88.87%
cyclist	87.83%	92.97%	94.71%	93.84%
Micro	90.06%	90.06%	90.06%	90.06%

**Table 6:** Neural Network Results—based on grid search (Test Dataset).

Class	Accuracy (%)	Precision (%)	Recall (%)	F1 Score (%)
car	87.66%	87.00%	87.47%	87.23%
pedestrian	94.59%	90.01%	87.66%	88.82%
cyclist	87.47%	92.68%	94.59%	93.62%
Micro	90.00%	89.91%	89.91%	89.91%

**Table 7:** Neural Network Results—based on genetic algorithm (Validation Dataset).

Class	Accuracy (%)	Precision (%)	Recall (%)	F1 Score (%)
car	97.93%	97.81%	97.93%	97.87%
pedestrian	98.26%	95.51%	98.26%	96.86%
cyclist	96.03%	99.00%	96.03%	97.49%
Micro	97.40%	97.41%	97.41%	97.41%

**Table 8:** Neural Network Results—based on genetic algorithm (Test Dataset).

Class	Accuracy (%)	Precision (%)	Recall (%)	F1 Score (%)
car	97.88%	97.79%	97.88%	97.84%
pedestrian	98.28%	95.57%	98.28%	96.90%
cyclist	96.09%	98.97%	96.09%	97.50%
Micro	97.42%	97.41%	97.41%	97.41%

**Table 9:** Different hyperparameter combinations (Validation part)—ablation study for the genetic algorithm. The best results are shown in bold.

Hyperparameters (Batch, Epochs, Opt, Lr, Hidden Units, Layers)	Balanced Acc.	Precision	Recall	F1
[256, 50, 0, 0.001, 30, 9]	**0.9741**	**0.9744**	**0.9740**	**0.9742**
[256, 50, 0, 0.001, 30, 12]	0.9736	0.9740	0.9736	0.9738
[64, 50, 0, 0.001, 30, 9]	0.9732	0.9736	0.9732	0.9734
[256, 50, 0, 0.001, 30, 10]	0.9731	0.9735	0.9731	0.9733
[32, 100, 0, 0.001, 10, 14]	0.9053	0.9064	0.9053	0.9059
[64, 90, 1, 0.0001, 10, 20]	0.8821	0.8858	0.8821	0.8839
[32, 90, 0, 0.00001, 18, 20]	0.8430	0.8623	0.8430	0.8525
[64, 50, 0, 0.00001, 12, 20]	0.8158	0.8309	0.8158	0.8233
[32, 80, 0, 0.01, 12, 14]	0.5888	0.4506	0.5888	0.5109

**Table 10:** Runtime, FLOPs, Memory Usage and Number of Parameters.

Metric	Grid Search Model	Genetic Algorithm Model
Parameters	443	8043
Memory usage (mb)	0.00196	0.03281
Flops	878	15,828
Batch size	1	1
Avg inference time (ms)	59.13	53.55
Std. Deviation (ms)	2.94	1.56
Min time (ms)	55.70	51.50
Max time (ms)	64.66	56.11
Avg throughput (samples/s)	16.95	18.69
Latency per sample (ms)	59.13	53.55
Batch size	256	256
Avg inference time (ms)	59.97	58.37
Std. Deviation (ms)	5.37	1.68
Min time (ms)	54.99	56.57
Max time (ms)	71.76	60.59
Avg throughput (samples/s)	4297.19	4389.25
Latency per sample (ms)	0.23	0.23

## Data Availability

The data presented in this study is available upon request from the corresponding authors. The data is not publicly available due to privacy concerns.
